# Successful treatment of refractory complete separation of an esophagojejunal anastomosis after laparoscopic total gastrectomy: a case report

**DOI:** 10.1186/s13104-017-2589-6

**Published:** 2017-07-11

**Authors:** Shinichi Oka, Shinichi Sakuramoto, Motohiro Chuman, Kenichi Aratani, Mitsuo Wakata, Yutaka Miyawaki, Hisashi Gunji, Hiroshi Sato, Koujun Okamoto, Shigeki Yamaguchi, Isamu Koyama

**Affiliations:** Department of Gastroenterological Surgery, Saitama Medical University International Medical Center, 1397-1 Yamane, Hidaka, Saitama 350-1298 Japan

**Keywords:** Anastomotic leakage, Esophagojejunal anastomosis, Laparoscopic total gastrectomy

## Abstract

**Background:**

Anastomotic leakage after total gastrectomy occurs despite improvements in surgical techniques and patient management. Although many cases of dehiscence can be managed non-operatively, major leakage requires a second surgery and can potentially lead to death. Therefore, accurate and immediate diagnosis and treatment are essential.

**Case presentation:**

In this report, we describe a 66-year-old Japanese man who was diagnosed with a complete separation of an esophagojejunal anastomosis after laparoscopic total gastrectomy with oral contrast radiography using Gastrografin^®^. The severe complication was successfully treated by re-anastomosis after two emergency drainage surgeries. After the second surgery, the esophageal end formed a fistula with the jejunum, but balloon dilation failed to open the fistula. Therefore, oral ingestion and conservative treatment were considered unsuitable, and we performed esophagojejunal re-anastomosis 7 months after the first surgery. At a follow-up examination 2 years after re-anastomosis, the patient weighed 47 kg, and his ingestion had recovered to 80% of that before surgery.

**Conclusions:**

Complete separation of an esophagojejunal anastomosis is a rare but severe complication of total gastrectomy. Therefore, we consider that once separation is diagnosed, aggressive and urgent re-operation and effective drainage are useful. Moreover, it is necessary to take great care to minimize the operative morbidity associated with esophagojejunal anastomosis.

## Background

The incidence of anastomotic leakage after total gastrectomy is 8.0–14.5% [1–4], and these levels persist despite improvements in surgical techniques and patient management. Although many cases of anastomotic leakage are minor and can be cured by non-operative treatments, such as fasting or hyperalimentation, major leakage occasionally requires a second surgery and can even lead to death [5]. Therefore, accurate and immediate diagnosis and treatment are essential. We searched PubMed, limiting our search to articles published in English during the last 20 years, but could not find any literature reports of dehiscence with complete separation after total gastrectomy. In previous reports, major leakage often indicated that the esophageal end and the elevated jejunal end were partially separated; however, in the present case, the ends were completely separated (by approximately 5 cm). Therefore, the present case appears to be very rare and involved potentially fatal complications. Here, we report a case of a complete separation of an esophagojejunal anastomosis after laparoscopic total gastrectomy (LTG), which we successfully treated by re-anastomosis after two emergency drainage surgeries.

## Case presentation

**Fig. 1:**
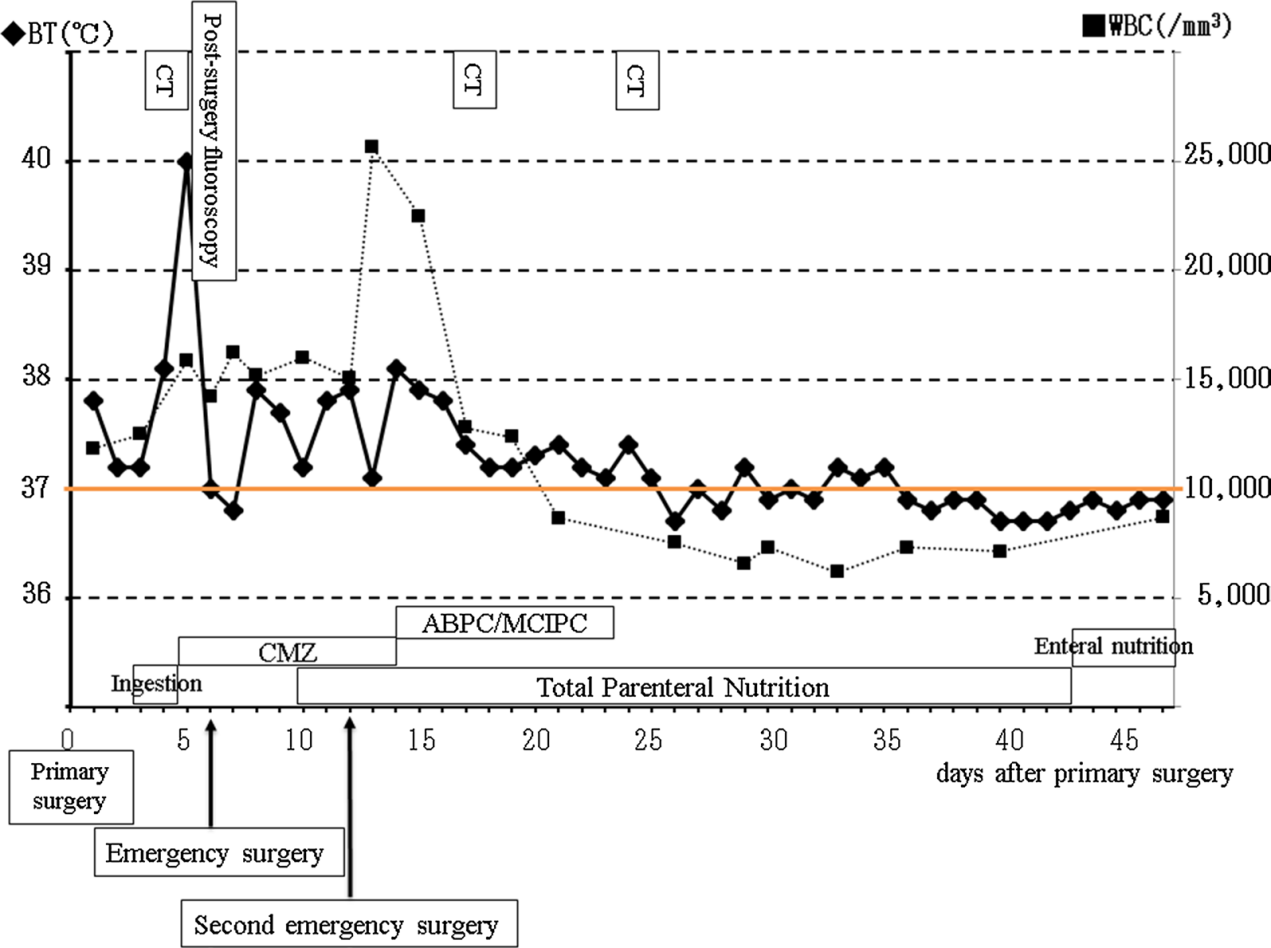
Clinical course of the patient. The patient’s clinical symptoms resolved, including his abdominal pain and fever, and his blood data improved (his white blood cell count was 8670/mm^3^ on day 7 after the second surgery). *ABPC* aminobenzylpenicillin, *BT* body temperature, *CMZ* cefmetazole, *CT* computed tomography, *MCIPC* cloxacillin, *WBC* white blood cell

**Fig. 2:**
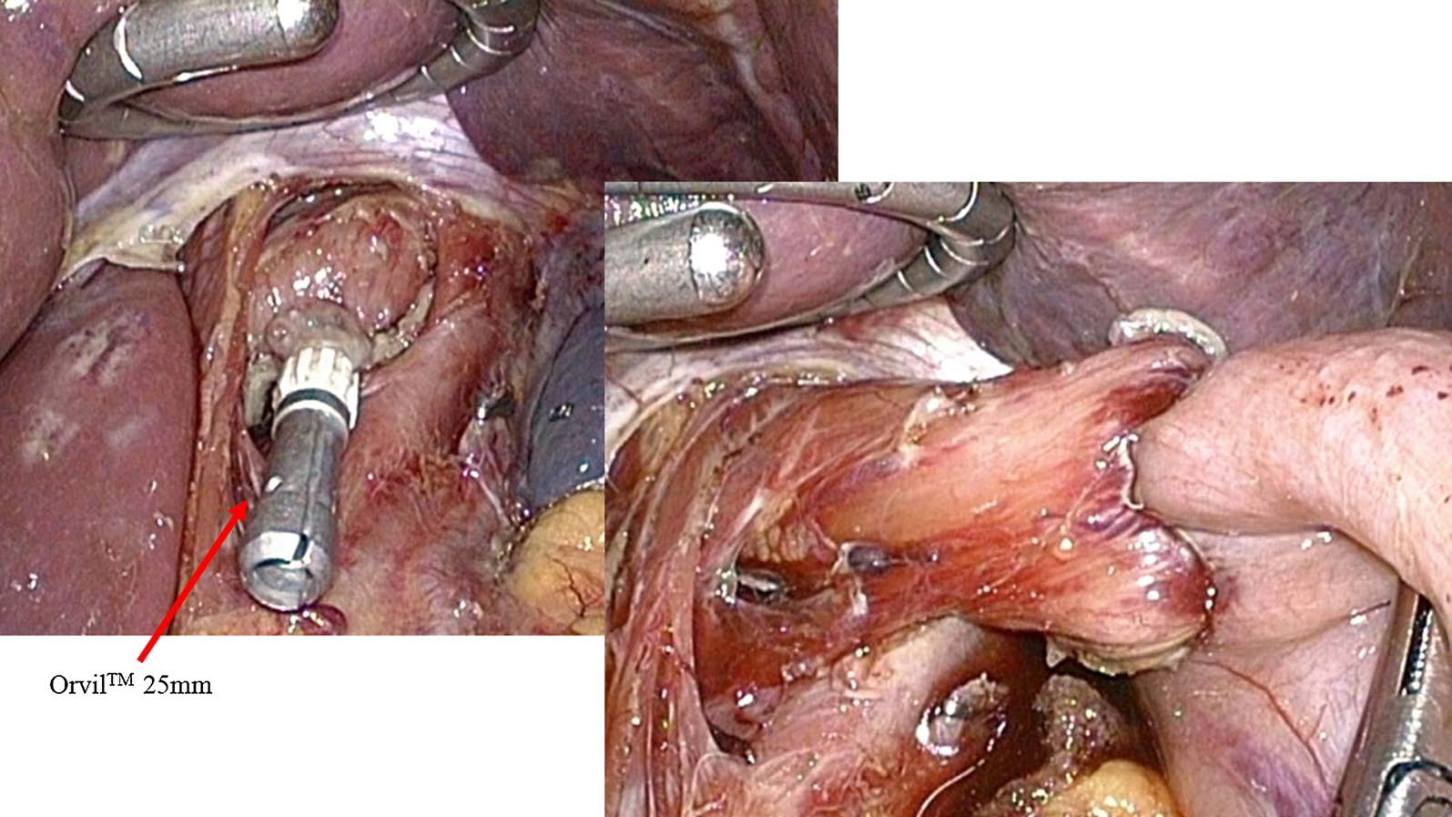
Esophagojejunostomy using a transoral anvil. A transoral anvil (OrVil™ 25 mm) was used for esophagojejunostomy. No vascular or organ injury occurred during the surgery

A 66-year-old Japanese man underwent LTG with D1 plus lymph node dissection and Roux-en-Y reconstruction for gastric cancer. His treatment course is summarized in Fig. 1. At hospitalization, his nutritional status, glucose tolerance, liver function, and lipid metabolism were all within normal limits; therefore, elective LTG was performed. Esophageal transection was performed with an endoscopic linear stapler. Then, a transorally inserted anvil (OrVil™; Covidien, Mansfield, MA, USA) was introduced into the esophagus. The operator indicated that the OrVil™ tube had reached the esophageal stump and made a small hole in the center of the esophageal stump with scissors. The tube was then extracted through the hole until the anvil reached the esophageal stump. The tube was then disconnected from the anvil by cutting the connecting thread and was removed from the abdominal cavity. Next, the jejunum was separated with a linear stapler approximately 25 cm from the Treitz ligament (Fig. 2). The elevated jejunum was retrocolically elevated. Esophagojejunostomy was then performed using a 25-mm EEA-XL stapler (Covidien, Mansfield, MA, USA). No evidence of vascular or organ injury was observed. Moreover, an intraoperative air leak test performed by immersion of the anastomosis in saline solution was performed at the end of the stapling, and no bubble escaped from the anastomosis. The operative time was 4 h 23 min, and minimal bleeding occurred.

The pathological findings were as follows: L, Post, Type 0–IIb, 54 × 36 mm, por 2 > sig, pT1a (M), UL (+), ly0, v0, pN0 (0/47), pPM0, pDM0, pStage IA.

**Fig. 3:**
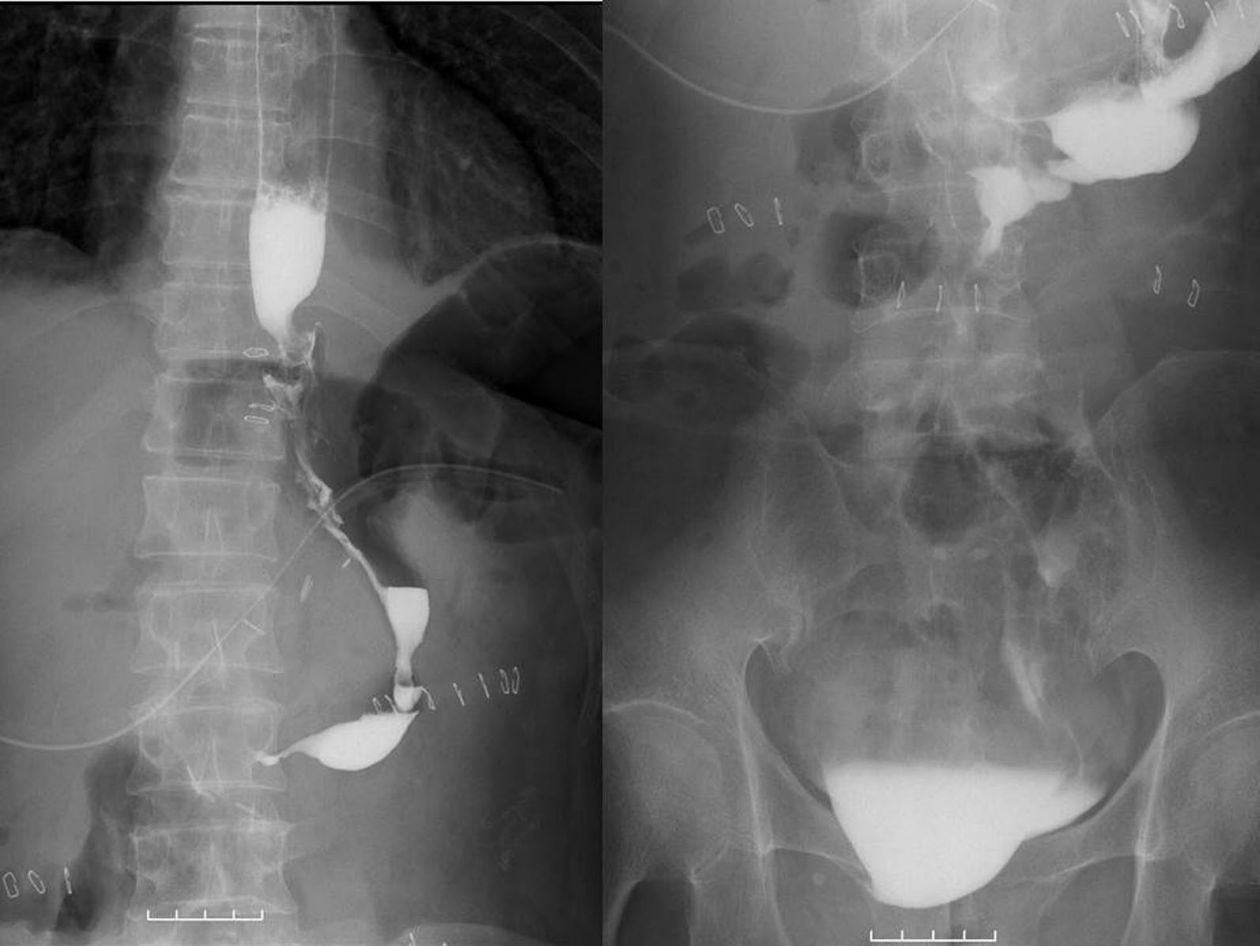
Oral contrast radiography. Gastrografin^®^ collections were evident, suggesting dehiscence

Following our usual clinical pathway, the patient was mobile by day 1 after surgery, drinking water on day 2, and had started a liquid diet by day 3. Although he developed a fever of approximately 38 °C on day 3, no associated abdominal pain was reported, and the drainage fluid was yellow and clear. However, on day 5, the patient complained of sudden abdominal pain while walking, and the drainage fluid had become turbid. Therefore, we stopped the diet immediately. On a subsequent abdominal computed tomography (CT) scan, no effusion was found near the esophagojejunal anastomosis or under the diaphragm; therefore, we concluded that the drainage was sufficient and that the leakage was minor. Thus, we began antibiotic medication. Despite our intervention, the abdominal pain continued to worsen, and the patient could not walk; therefore, we performed oral contrast radiography with Gastrografin^®^ on day 6. This procedure showed that the contrast medium immediately leaked from the esophageal end of the anastomosis to the pelvic space, and we diagnosed complete separation of the anastomosis (Fig. 3). We considered lavage and drainage necessary and performed emergency surgery on the same day.

**Fig. 4:**
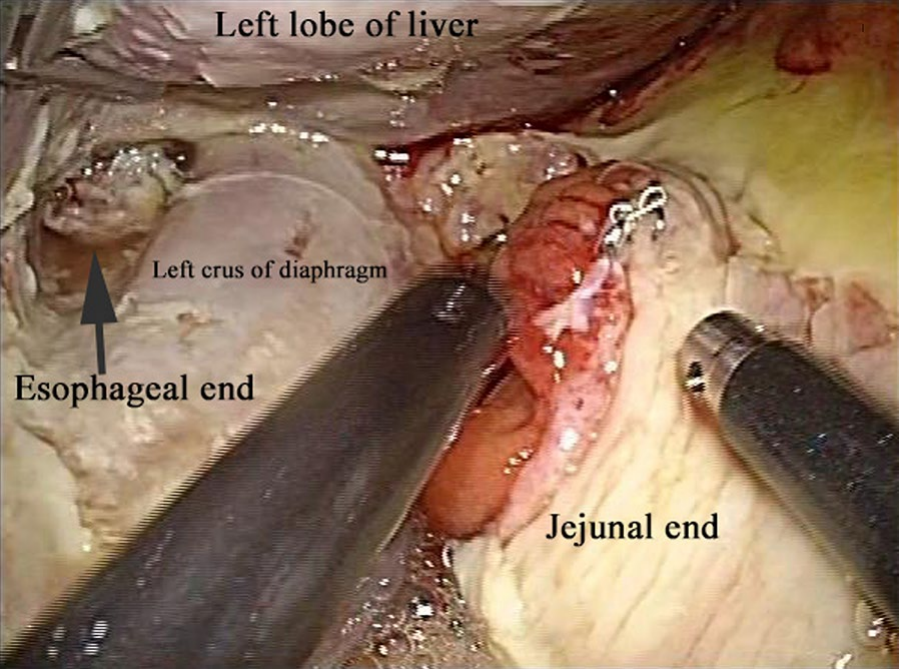
The refractory total separation of the esophagojejunal anastomosis region. Laparoscopy revealed saliva and contaminant ascites causing a thick white collection around the esophagojejunal anastomosis. The esophagojejunal anastomosis was completely separated, with the ends approximately 5 cm apart, and the remaining staples formed a B shape

During emergency laparoscopy, saliva and contaminated ascites were observed to cause a thick white collection around the esophagojejunal anastomosis. Complete separation of the esophagojejunal anastomosis was confirmed after irrigating the upper abdomen; the ends were separated by approximately 5 cm, and the remaining staples formed a B shape (Fig. 4). No evidence of color change was present in any layer of the anastomosis at the site of the elevated jejunum, suggesting that this dehiscence was unlikely due to insufficient blood perfusion. Re-anastomosis was considered too difficult in the presence of ongoing inflammation; therefore, the esophageal and jejunal openings were closed by sutures to prevent the leakage of digestive fluid and saliva. The surgery was completed by leaving drainage tubes in the esophageal end, in both sub-diaphragmatic spaces, and in the pouch of Douglas.

**Fig. 5:**
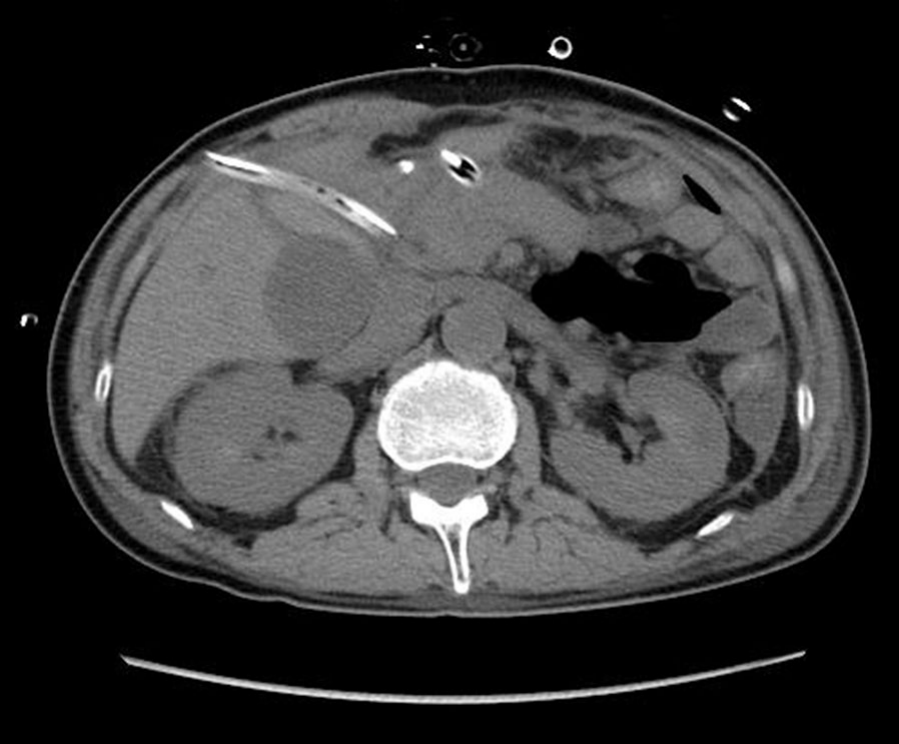
Computed tomography 6 days after the first emergency surgery. Fluid accumulation could be observed in the anterior area of the inferior duodenal flexure together with peritoneal hypertrophy of the whole abdominal area

On day 12, 6 days after the first emergency surgery, a CT scan suggested continued peritonitis with evidence of diffuse peritoneal hypertrophy and fluid accumulation in the anterior area of the inferior duodenal flexure (Fig. 5). Therefore, a second emergency surgery was performed. We found an accumulation of ascites near the esophageal hiatus and the elevated jejunum stump. Although the esophageal end had been sutured during the previous surgery, the sutures had failed to hold, and the stump was open, with the esophagus pulled into the mediastinum, allowing saliva to leak into the abdominal cavity. However, we did not re-close the esophageal end but instead opted to leave a tube in situ to drain saliva. The elevated jejunal end was also open, but we closed this end firmly using serosa-muscle-layer sutures that introverted the mucosa. We also created an intestinal fistula to permit nutrition, which was formed at 20 cm on the anal side from the Y portion.

**Fig. 6:**
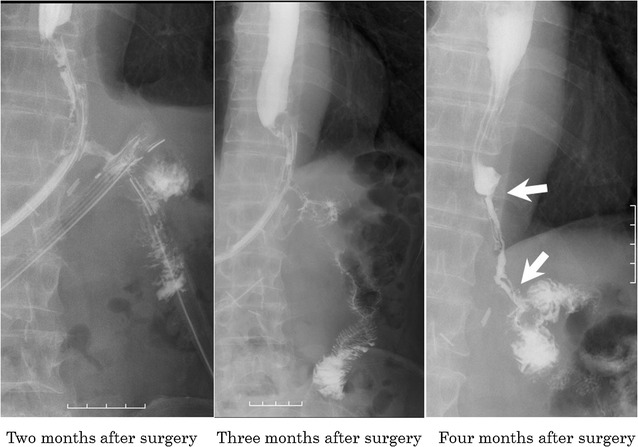
Oral contrast radiography revealed a fistula. Contrast medium was observed leaking from the dehiscence into the drainage tube after 2 and 3 months, but this had stopped by 4 months postoperatively, and the contrast medium was observed to flow from the esophagus to the jejunum, suggesting that a fistula had formed between the two ends

**Fig. 7:**
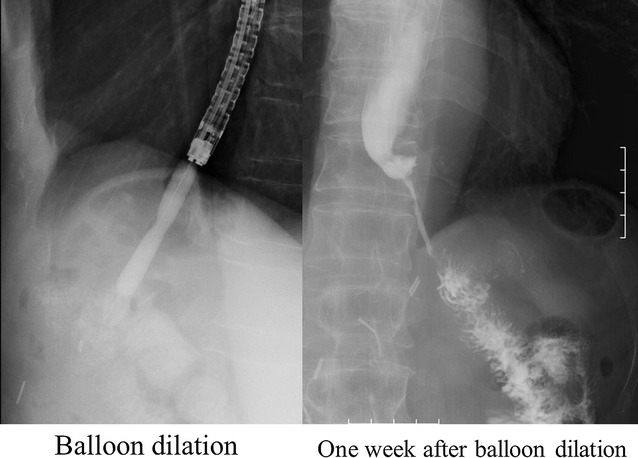
Balloon dilatation was attempted to widen the fistula. The size of the fistula was approximately 6 cm in length and 0.2 cm in diameter when noted. Therefore, to avoid further invasive surgery, we first opted to repeat dilation every 1 or 2 weeks. Although we successfully increased the diameter of the fistula to 0.8 cm, the patient remained unable to ingest food

After the second surgery, the patient’s clinical symptoms resolved, including his abdominal pain and fever, and an improvement in his blood data (white blood cell count, 8670/mm^3^ on day 7 after the second surgery) was noted. We then started enteral nutrition on day 43 (Fig. 1), and although oral contrast radiography showed leakage of the contrast medium from the dehiscence into the drainage tube at 2 and 3 months after the surgery, it had stopped by 4 months postoperatively. By this time, contrast medium was observed to flow from the esophageal end to the elevated jejunum, suggesting the development of a fistula (Fig. 6). The fistula measured approximately 6 cm in length and 0.2 cm in diameter. Given the location and path of the fistula, we performed balloon dilation (Fig. 7) as a non-operative treatment every 1 or 2 weeks. Although we dilated the fistula to 0.8 cm in diameter, oral intake was not achieved, and we concluded that non-operative treatment alone would not be sufficient.

**Fig. 8:**
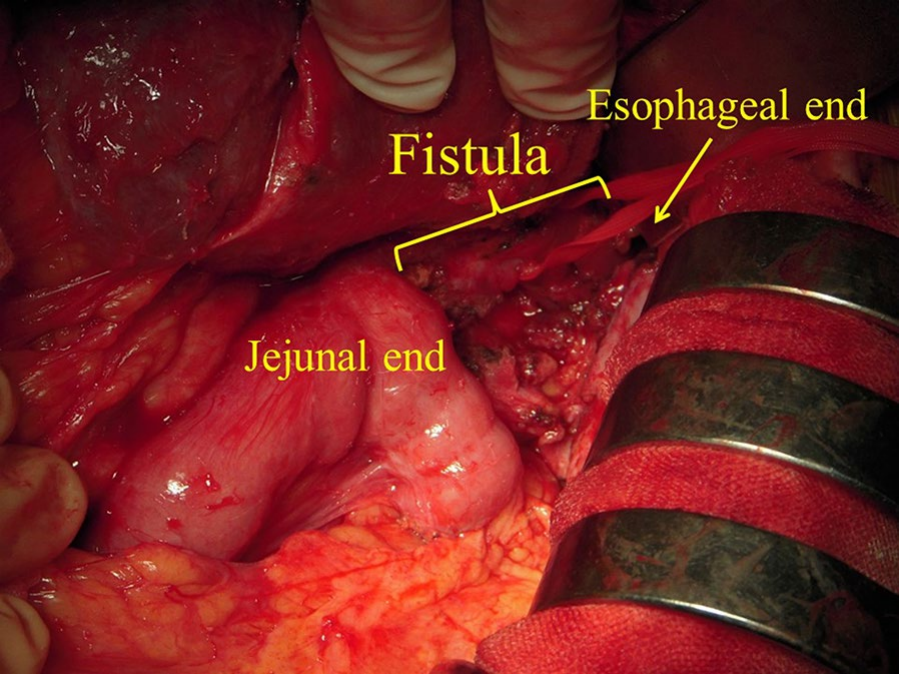
Surgical findings at re-anastomosis. We performed esophagojejunal re-anastomosis at 7 months after the last surgery. During surgery, a complete fistula was found between the ends of the esophagus and the jejunum

Due to the lack of a satisfactory outcome from non-operative treatment, we chose to perform esophagojejunal re-anastomosis after 7 months. A complete fistula was observed between the oesophagus and the jejunum (Fig. 8) at the point where complete separation of the esophagojejunal anastomosis had previously been observed (Fig. 4). During surgery, we exposed and taped the fistula and resected a 6-cm portion; the esophageal end showed inflammation-related hypertrophy and sclerosis, which were pulling it into the mediastinum; therefore, we dissected the esophagus beyond the dehiscence-induced inflammation and resected the sclerotic esophageal tissue using a linear stapler. We also resected the elevated jejunum stump, including the previous anastomosis site, using a linear stapler. After confirming that both the esophagus and elevated jejunum had sufficient blood perfusion and were not tense or twisted, we created a side-to-side esophagojejunal anastomosis using an Endo-GIA stapler (Covidien, Mansfield, MA, USA), overlapping both ends on the left side of the esophagus, again using an Endo-GIA stapler.

**Fig. 9:**
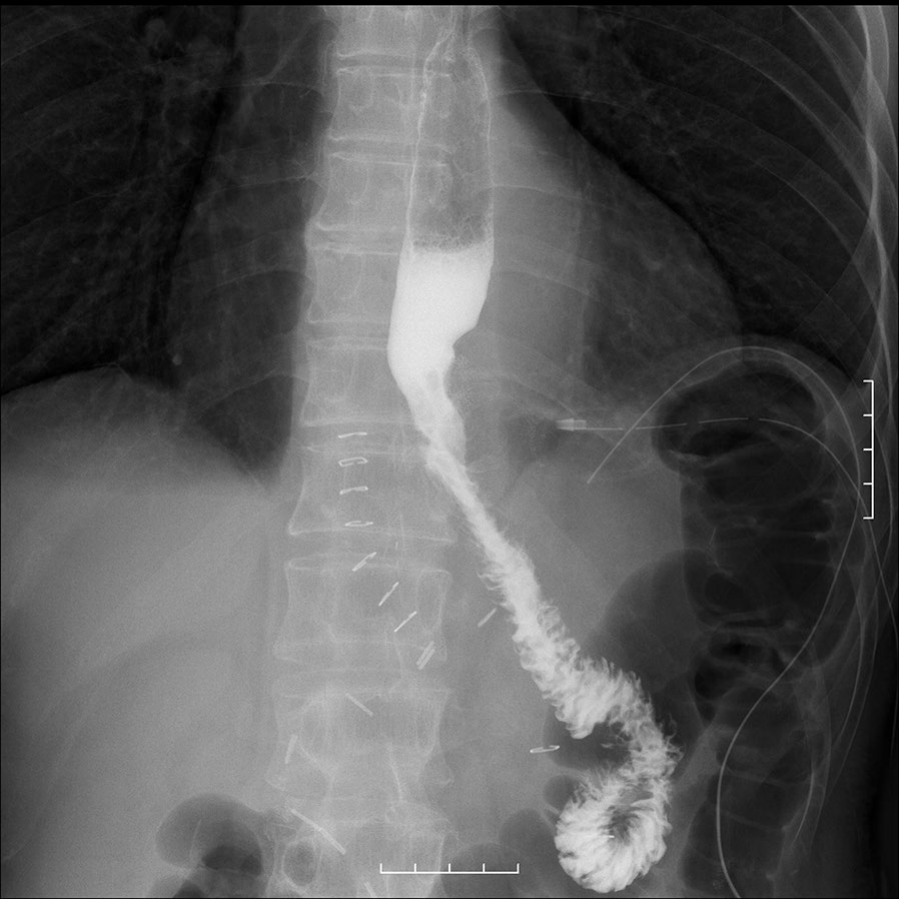
Oral contrast radiography 4 days after re-anastomosis. The outflow of contrast medium was good, and no stenosis or reflux occurred

Oral contrast radiography performed 4 days after the re-anastomosis showed no signs of dehiscence (Fig. 9); therefore, oral ingestion was started 8 days after surgery. However, the patient developed an abdominal abscess secondary to a wound infection on day 9 after the re-anastomosis, progressing to endocarditis, cardiac tamponade, and bilateral pleural effusions by day 18, which caused his general condition to worsen. Because of the endocarditis and cardiac tamponade, it was suggested that extremely severe inflammation had been caused by partial injury of the pericardium during esophageal resection in the re-anastomosis. We provided ultrasound- and CT-guided abdominal, pericardial, and thoracic drainage, together with antibiotic treatment, and the patient’s general condition improved. Later, ingestion was started on day 28 after the re-anastomosis, and the patient was discharged 8 months after the initial surgery, 47 days after the re-anastomosis.

**Fig. 10:**
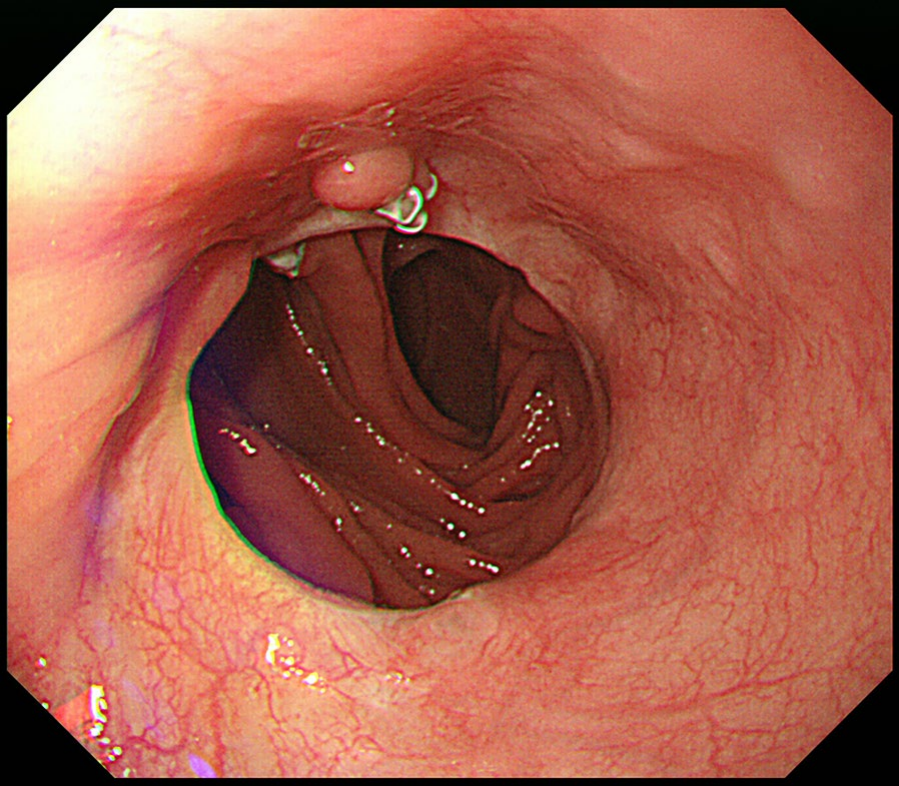
Gastrointestinal endoscopy 1 year after re-anastomosis. Endoscopy at 1 year after re-anastomosis showed no stenosis, food obstruction, or reflux esophagitis

Endoscopic examination at 1 year after re-anastomosis showed no stenosis at the anastomosis site, food obstruction, or reflux esophagitis (Fig. 10). Approximately 2 years after discharge, the patient weighed 47 kg, and his ingestion had recovered to 80% of that before surgery. The patient continues to enjoy sporting activities as he previously did and attends our outpatient clinic every 3 months.

## Discussion

Although surgical techniques have improved, dehiscence still occurs with a certain frequency, with postoperative esophagojejunal anastomotic leakage occurring at a rate of 0.5–11.0% [1–12]. According to a report of the Japanese National Clinical Database for digestive surgery, the incidence of anastomotic leakage after total gastrectomy was 4.4% (881 of 20,011 cases) in 2011 [12]. The details (whether open or laparoscopic) were not listed in this report, but several studies have indicated that anastomotic leakage is observed significantly more often in laparoscopic surgery than in open total gastrectomy or laparoscopic distal gastrectomy [12–14]. However, conflicting data exist [15], and several studies have reported that the surgeon’s experience of LTG is related to the rate of anastomosis-related complications [16–20]. According to Jeong et al., multivariate analysis showed that postoperative morbidity significantly differed according to the surgeon’s experience (fewer than 45 cases) [19].

Several devices, such as circular staplers or linear staplers, are used by surgeons for various methods including the OrVil™ method [21], overlap method [22], and the functional end-to-end anastomosis method [23]. We are attempting to standardize the techniques used for esophagojejunal anastomosis using the OrVil™ method for LTG [21]. In our case, the surgeon’s experience of LTG included approximately 50 cases. Furthermore, we performed esophagojejunostomy using OrVil™ as usual in this case. This level of experience may be sufficient. However, it may be necessary to take great care to minimize operative morbidity associated with esophagojejunal anastomosis.

Symptoms of anastomotic leakage include abdominal pain, peritoneal irritation, backache, lumbago, and continuous remittent fever over 38 °C, accompanied by leukocytosis and an elevated C-reactive protein level [24]. Diagnosis is established based on these symptoms and laboratory data, together with findings on gastric fluoroscopy and abdominal CT examinations [25]. However, sometimes anastomotic leakage cannot be diagnosed clearly from gastric fluoroscopy alone, and evaluations of the distribution of an intraperitoneal abscess or the effectiveness of drainage can be difficult [26].

In the present case, a CT scan was performed to evaluate whether any intraperitoneal fluid accumulation had occurred. No effusion was found near the esophagojejunal anastomosis or under the diaphragm; however, oral contrast radiography with Gastrografin^®^ showed that the contrast medium was leaking from the esophageal end of the anastomosis to the pelvic space, and we diagnosed a complete separation of the anastomosis. Therefore, we consider that both fluoroscopic examination and CT scans should be performed immediately when dehiscence is suspected, even when no contrast medium drains from drainage tubes.

Anastomotic leakage may simply affect quality of life but can also cause abdominal pain, fever, and difficulties with ingestion that require the patient to be hospitalized for prolonged periods [3, 5, 6]. Minor leakage rarely becomes severe, whereas major leakage sometimes leads to peritonitis accompanied by sepsis and multiple organ failure or death. When the dehiscence is major, urgent re-operation is sometimes needed, but the reported mortality rate with re-laparotomy is as high as 37.5% [1].

In our case, the anastomosed ends of the esophagus and elevated jejunum were completely separated. We considered lavage and drainage of the abdominal cavity to be necessary and performed an emergency surgery. Moreover, an additional emergency surgery was required to suppress inflammation. After two emergency drainage surgeries, we finally performed re-anastomosis surgery successfully despite the complete separation of the anastomosis. In our case, we consider that infection control and aggressive urgent re-operation were the most important and useful procedures adopted.

Despite developing a complete anastomotic separation, which is an extremely severe complication, our patient was eventually treated successfully. However, it is noteworthy that treatment plans were changed regularly during the treatment course based on the patient’s general condition and nutritional status, as well as the development of wound infection and an intraperitoneal abscess. Consequently, re-anastomosis was not performed until 7 months later. We have now observed many times that the elevated jejunum can reach the esophageal stump in a natural state without any twisting. We consider that this procedure can prevent excessive tension. In addition, we now add an extra three or four non-absorbent sutures (4-0) through all layers of the anastomosis to reinforce the anastomosis.

## Conclusions

In conclusion, we present a rare case of the complete separation of an esophagojejunal anastomosis after LTG, which we successfully treated by re-anastomosis after two emergency drainage surgeries.

The complete separation of an esophagojejunal anastomosis is a rare but severe complication. Therefore, once such a case is diagnosed, aggressive urgent re-operation and effective drainage are required. We believe that this is the most important and useful approach. Moreover, it may be necessary to take additional care to minimize operative morbidity associated with esophagojejunal anastomosis.

## Abbreviations

ABPC: aminobenzylpenicillin
BT: body temperature
CMZ: cefmetazole
CT: computed tomography
LTG: laparoscopic total gastrectomy
MCIPC: cloxacillin
WBC: white blood cell
